# Progress in Obstetrics

**Published:** 1903-07-11

**Authors:** 


### Progress in Obstetrics.

**Puerperal Eclampsia.**—W. E. Fothergill,[1] in a paper on the ætiology and treatment of eclampsia, discusses the various theories as to its cause, and reviews the modern treatment of the disease. He draws attention to the importance of prophylactic treatment, and says that a condition of toxæmia, which may end in eclampsia, can often be diagnosed long before the occurrence of convulsions, and that appropriate treatment undertaken at this stage may exert such a favourable influence as to admit of the termination of pregnancy at term without disaster. Among the symptoms which point to the existence of the pre-eclamptic state may be mentioned vomiting, constipation, headache, nervous irritability, disturbances of vision, and abnormal pigmentation. The appearance of albumin in the urine, and the occurrence of œdema are still more definite signs. The prophylactic treatment generally adopted consists of rest in bed, milk diet, purgation, and the washing out of the lower bowel with copious injections of warm water. Recently the administration of some preparation of the thyroid gland has been strongly recommended as a prophylactic measure. On the appearance of the above-mentioned symptoms, even if there is no albumin in the urine, 5 grains of thyroid extract should be given three times a day. In some of the reported cases the symptoms of toxæmia disappeared under the thyroid treatment, but returned repeatedly when the treatment was stopped, and again vanished when it was recommenced. If these pre-eclamptic symptoms are unrelieved by treatment, labour should be induced. While the induction of labour is recognised as allowable in the prophylaxis of eclampsia, the question of operative obstetric interference in the presence of convulsions remains in doubt. According to the views of one party, the treatment should be limited to gently accelerating the progress of labour in cases in which it has already begun, while according to the other, obstetric treatment is all-important and the uterus must be emptied as soon as possible, either by incision of the cervix, dilatation of the cervix with Bossi's instrument, or by Cæsarian section. With regard to the medical treatment of actual eclampsia certain pronounced changes have occurred during recent years. Thus chloroform is much less used than was previously the case. Croton oil and other purgatives have been dropped in favour of lavage of the rectum and lower bowel, while pilocarpine has been displaced by the useful and perfectly harmless wet pack. Morphia has rather taken the place of chloral and bromide. Bleeding has become more common. The most striking feature in the recent history of eclampsia is the rapidity with which the treatment by subcutaneous injection of saline solution has become general. The credit for this belongs in a great measure to Jardine, of Glasgow. Since this treatment has been adopted in the Glasgow Maternity Hospital, the mortality of eclampsia in that institution has been only 17 per cent., whereas during the previous 15 years it was 47 per cent. The point insisted on by Jardine is that, in the treatment of eclampsia, the main object is to restore the renal function, and this indication is well met by saline infusion. Lastly, the administration of thyroid substance has been recommended not only as a method of prophylactic treatment, but also for the treatment of actual eclampsia. Nicholson holds that the poisons which cause eclampsia are also powerful vaso-constrictors, and act more particularly on the renal vessels, thus leading to suppression of urine. Thyroid substance is proved to be a powerful, rapid, and permanent vaso dilator, and if in eclampsia it be administered so as to produce symptoms of thyroid intoxication, it appears that the spasm of the renal vessels is relaxed and diuresis follows at once. Ten or fifteen grains of the dry extract may be given by the mouth and repeated if necessary every hour; and if the patient cannot swallow, similar doses of the liquor thyroidei must be injected hypodermically. Nicholson also points out that the success of other remedies may depend upon their action in relaxing spasm of the renal arterioles and lowering blood pressure, for morphia given in large doses acts as a prompt and powerful vaso-dilator. Free bleeding lowers blood-pressure, and saline solution introduced into the circulation has the same effect. J. W. Ballantyne[2] reports three cases of eclampsia in which he employed Bossi's dilator. In all of them morphia was given and in one saline transfusion. He thinks that if we accept the principle of early completion of labour in eclampsia, Bossi's instrument enables us to do so more quickly than by any other means.

**Pregnancy in Connection with Chronic Heart Disease.**—Cuthbert E. Gibbs,[3] in an instructive paper on this subject, says that the fundamental rules as regards treatment are (1) to promote the general health of the patient, (2) to avoid unduly taxing the heart, and (3) to furnish the heart with as pure a blood supply as possible. In compensated cases the routine of everyday life should be carried out with the view of improving the general tone of the system. Diet permits of great latitude during the early months, but care is necessary in order to avoid the dyspeptic troubles so common in pregnant women, flatulent distension of the stomach proving a source of great embarrassment to the heart. In cases of mitral stenosis he advises in the later weeks a mixture containing the following:—L. Nucis vom m v., sp. æther. m xv. sp. ammon. aromat m xv., liq. trinitrini m $\frac{3}{4}$, three times a day. Acute paroxysm of dyspnœa should be treated with oxygen inhalations, and hypodermic injections of strychnine and æther, in order to tide the patient over the crisis. For sleepnessness he recommends opium, or chloretone. With regard to the question of inducing labour, he thinks that the majority of cases are better left alone, but if premature labour has to be induced, it should be done before the cardiac symptoms become urgent and the patient worn out by the long struggle. If this opportunity be lost the patient stands a better chance if left to nature. In cases of hydramnios when the upward displacement of the diaphragm largely increases the urgency of the symptoms, labour should always be induced. During labour the heart should be relieved of as much strain as possible. In the first stage bearing down should be avoided, and when the breathing is much embarrassed oxygen inhalations should be administered from time to time. If the heart shows any sign of flagging ether or strychnine should be administered alternately. Artificial dilatation of the os must be resorted to if necessary, and in any case an anæsthetic should be administered and the forceps used as soon as the os is fully dilated. It is never advisable to hurry the removal of the placenta, but rather to allow free hæmorrhage to take place, for these cases die far oftener from too little hæmorrhage than from too much. Absolute rest in the horizontal position should be insisted on for at least the first week after labour, and the patient should not be allowed to sit up for three or four weeks.

**Puerperal Septicæmia.**—Charles S. White[4] advocates the use of antistreptococcic serum in the treatment of puerperal septicæmia, and records a case in which marked improvement took place from the time it was employed, and this after the usual treatment had failed to check the progress of the disease. He thinks we are justified in employing this serum in all cases of sepsis due to the streptococcus. Dangerous or alarming symptoms following its use rarely occur, and although it may fail in some instances, remarkable achievements have been recorded. Leopold[5] considers that further experience is required to show to what extent the injection of antistreptococcic serum might be depended on. He thinks that perhaps in the gravest cases removal of the suppurating venous trunks might in the future be productive of good results. There is still considerable difference of opinion as to the value of hysterectomy in cases of puerperal septicæmia. Fehling,[6] from the study of a number of cases draws the following conclusions:—(1) In generalised infection removal of the uterus has no chance of success and should not be performed. (2) Hysterectomy is only indicated if the focus of infection or intoxication is limited to the uterus, as in cases of putrefaction of placenta, infection of a myoma, or retained products after abortion, and if it is impossible to remove these by other means. (3) In puerperal metro-phlebitis hysterectomy may be useful if accompanied by ligature of the thrombosed uterine and ovarian veins.

[1] Practitioner, Feb. 1903. [2] Brit. Med. Jour., Feb. 21, 1903. [3] The Clin. Jour., Jan. 21, 1903. [4] Med. Rec., Jan. 10, 1903. [5] Lancet, Nov. 15, 1902. [6] Med. Chron., Dec. 1902.

